# Infrared Thermal Imaging (ITI), a Non‐invasive Window Into Early Emotion Regulation: A Systematic Review

**DOI:** 10.1002/dev.70071

**Published:** 2025-08-12

**Authors:** Sarah Nazzari, Miriam Paola Pili, Ekin Çelik, Samuele Lucchin, Livio Provenzi

**Affiliations:** ^1^ Department of Brain and Behavioral Sciences University of Pavia Pavia Italy; ^2^ Developmental Psychobiology Lab IRCCS Mondino Foundation Pavia Italy

**Keywords:** autonomic nervous system, child, development, emotion regulation, infrared thermal imaging, skin temperature, stress

## Abstract

Investigating early emotion regulation abilities is crucial as they are key predictors of future socio‐emotional development. Infrared thermal imaging (ITI) is a promising non‐invasive technique for studying physiological regulation of socio‐emotional states in children in both ecological and controlled settings. Despite its potential, no review has summarized the current evidence in the field. We performed a systematic review following the PRISMA guidelines to analyze temperature changes in response to socio‐emotional stimuli in children aged 0–12 years. The search yielded 15 records, published between 1959 and 2023, including typically developing children (*n* = 13) and children with neurodevelopmental conditions (*n* = 2). The reviewed studies showed mixed results, with methodological quality ranging from weak to moderate. Temperature increases and decreases were reported across regions of interest, particularly in the face and hands, in response to negative and positive emotions elicited by face‐to‐face interactions and audio–visual stimuli. The limited evidence and methodological variability across studies prevent the identification of clear patterns in children's thermal responses to socio‐emotional stimuli. Further rigorous research is needed to validate ITI as a reliable tool for exploring socio‐emotional regulation in children with typical and atypical development.

## 1 Introduction

Emotions carry significant communicative value, starting from the earliest stages of human life (Ioannou et al. 2021). The development of socio‐emotional regulation is a fundamental aspect of typical and atypical development. It serves as a precursor of various dimensions of social functioning, such as social competence, positive relationships, empathy, etc. Effective emotion regulation is also considered a protective factor against internalizing and externalizing problems during childhood, supporting healthy adjustment in adulthood. Conversely, difficulties in developing adaptive regulation strategies early in life are linked to socio‐emotional and behavioral challenges and an increased risk of psychopathology (Atkinson et al. 2021; Cole et al. 2020; Crespo et al. 2017; Kim et al. 2014; Leerkes et al. 2009; Penela et al. 2015; Rawana et al. 2014; Thomas et al. 2017).

Regulating emotional states implies the ability to manage, adjust, suppress, or amplify emotions (Calkins and Fox 2002). It includes behaviors that can be deliberate or automatic, as well as conscious or unconscious (Cole et al. 2020). In the regulatory processes, including the emotional one, the autonomic nervous system (ANS) plays a significant role (Feldman 2007). The ANS consists of the parasympathetic nervous system (PNS) and the sympathetic nervous system (SNS), which are believed to work in complementary ways to help individuals respond and adapt to environmental challenges. The PNS is primarily active during restful states, maintaining homeostasis, while the SNS is engaged during perceived threats, triggering the fight—or—flight response by increasing heart rate and mobilizing metabolic resources. According to Porges’ polyvagal theory (Beauchaine 2001; Porges 2007a), the ability to temporarily disengage the PNS in mildly challenging situations represents an evolutionary advancement in arousal regulation. This adaptation enables individuals to address environmental demands without relying on the more energy—intensive activation of the SNS (Beauchaine 2001; Suurland et al. 2017). A perceived safe environment enables the activation of the PNS while suppressing the more evolutionarily primitive SNS. However, the extent to which the PNS and SNS respond to stress varies among individuals and may be influenced by early life experiences (Beauchaine 2001; Oosterman et al. 2010; Suurland et al. 2017).

Conventional gold standard physiological methods for assessing ANS function include electrocardiography and skin conductance measurements (Ioannou et al. 2014). These techniques required the use of contact sensors, limited movements, and high participants’ compliance, thus limiting the ecological validity of these measurements. This could be especially problematic with a very young population where the compliance cannot be guaranteed, and experimental manipulations could lead to biased assessment of infants' emotional states. Infrared thermal imaging (ITI) has been recently proposed as a non‐invasive method for investigating aspects of early parent—infant interaction, including the physiological co‐regulation of emotional states (Nazzari et al. 2024). ITI devices enable the examination of changes in ANS activity as reflected by variations in skin temperature in real time and in naturalistic settings. Thermal directional changes on the skin related to blood flow are the most widely explored thermal indices (Ioannou et al. 2014); blood flow transfers heat from the body's core to the skin and is regulated by vascular processes mainly controlled by the SNS. Vasoconstriction, primarily governed by the SNS, leads to a localized decrease in skin temperature, whereas vasodilation and gradual temperature increase are observed following SNS withdrawal (Donadio et al. 2006; Kosonogov et al. 2017). Most studies focused on measuring facial skin thermal variations (Ioannou et al. 2014), as the face can usually be easily recorded and is highly involved in social interaction. ITI has been found to be reliable when compared with simultaneous recordings from gold‐standard methods, such as ECG and skin conductance (e.g., Kuraoka and Nakamura 2011; Shastri et al. 2009). Furthermore, ITI can accurately capture psychophysiological arousal states and distinguish them from baseline conditions (Nhan and Chau 2010; Shastri et al. 2009). Lastly, due to its non‐invasive and contact‐free approach, ITI offers a unique opportunity to study emotion regulation skills in a more ecological and natural setting. Despite its potential, applications of ITI to the investigation of early emotion regulation remain limited. This is particularly noteworthy given that the early years of life are critical for the development of emotion regulation capacities, which are shaped by interactions with caregivers and the surrounding environment. By leveraging ITI, researchers could gain new insights into the underlying mechanisms of emotion regulation and the developmental changes that occur during infancy and early childhood. Moreover, ITI could serve as a valuable tool in identifying early indicators of atypical regulation patterns or vulnerabilities to psychopathology, thereby informing interventions aimed at supporting optimal development. This review aims to systematically examine available evidence in the field of ITI as applied to the study of early emotion regulation, while highlighting methodological limitations and insights for future research directions.

## 2 Methods

### 2.1 Literature Search

This systematic review was conducted from February 2024 to September 2024 following the Preferred Reporting Items for Systematic Review and Meta‐Analysis (PRISMA) guidelines (Liberati et al. 2009). A comprehensive literature search was carried out at two time points, including three different databases, specifically PubMed, Scopus, and Web of Science. While the first literature search was performed on February 5th, 2024, the second search was completed on March 19th, 2024. A very precise search string was decided [(infant* OR child* OR toddler* OR preschool*) AND (emotion* OR stress* OR regulation*) AND (“thermal imaging” OR “skin temperature” OR long‐infrared OR thermography*)] to catch the available studies in the literature to be conveyed in the review study without any restriction in terms of date of publication. In total, 1131 records were found: 479 on Scopus, 80 on PubMed, and 570 records on Web of Science.

### 2.2 Data Selection

**FIGURE 1:**
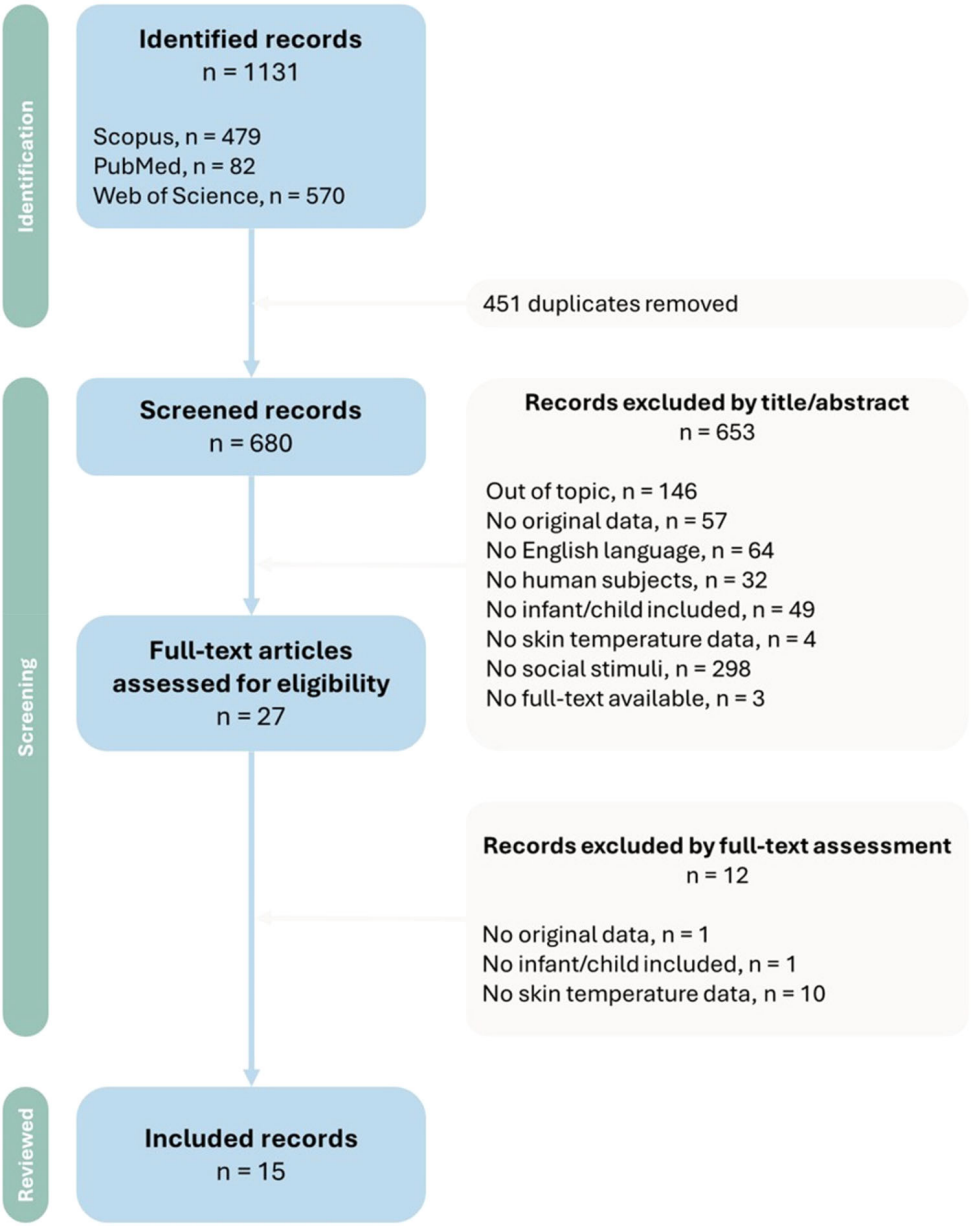
Flow diagram of study selection following PRISMA guidelines.

The retrieved 1131 records were checked for duplicates using Rayyan (Ouzzani et al. 2016). After the removal of 451 duplicates, 680 records remained. The screening of the remaining papers was carried out by two independent researchers (SL and EC), by consideration of titles, abstracts, and full text. We included only (1) original papers reporting empirical data, (2) in English, (3) on humans, (4) assessing thermal response to socio‐emotional stimuli in children, (5) aged between 0 and 12. We set the upper age limit at 12 years to capture the developmental period of childhood, prior to the onset of adolescence, which is characterized by significant neurobiological and socio‐emotional changes (Allen and Nelson 2018; Dong et al. 2021; Gunnar et al. 2009). This cutoff minimizes developmental heterogeneity and aligns with our focus on early emotion regulation processes, as well as with the age range most commonly targeted in pediatric ITI studies. Records were considered eligible if they were already published or available as online previews ahead of print. We deliberately excluded grey literature, such as conference proceedings or posters, to ensure that all included studies had undergone a rigorous peer‐review process, thereby prioritizing methodological quality and established findings within the scope of this review. We acknowledge that, while this approach enhances the robustness of our conclusions, it may have led to the omission of some preliminary or cutting‐edge findings. No previous reviews, theoretical pieces, viewpoints, letters, or posters were included. Figure 1 illustrates the flow chart of the systematic literature search and record selection following the PRISMA guidelines.

### 2.3 Data Abstracting and Synthesis

After screening the records, only 27 articles met the eligibility assessment. After reading the full texts, 12 works were further excluded for the following reasons: one study did not include the eligible age range, 10 studies did not measure skin temperature, and one was a duplicate of an already included study. With the remaining 15 articles, a data abstraction has been conducted to convey this information: (a) record information (i.e., authors, year of publication); (b) methodology (i.e., sample size, male‐female ratio of participants, control group [if any], confounders); (c) child characteristics (i.e., age of children, age range, type of clinical population [if any]); (d) social stimulus that was used in the study (i.e., type of social stimulus, a brief explanation of each); (e) type of emotion that was considered in the experiment (i.e., emotions of interest, nature of emotion); (f) physiological assessment (i.e., type of ITI camera, region[s] of interest, acquisition rate, Epoch duration); (g) key findings. Full‐text screening and data abstraction were conducted independently by two reviewers (SL and EC). To ensure consistency, a subset of studies (approximately 7.4%, *n* = 50) was reviewed by both reviewers, yielding a percent agreement of 98%. Discrepancies were resolved through discussion with a third author (MPP).

### 2.4 Assessment of Methodological Quality of Included Studies

Two reviewers (SL and EC) perform the assessment of the methodological quality of the included studies using the Effective Public Health Practice Project (EPHPP) Quality Assessment Tool for Quantitative Studies (Thomas et al. 2004). The EPHPP Quality Assessment Tool covers different sorts of research designs, such as randomized control trials and non‐randomized experiments (Thomas et al. 2004), and consists of six components (i.e., selection bias, design, confounders, blinding, data collection methods, and withdrawals and dropouts). Each component is rated either strong (1), moderate (2), or weak (3) and a global rating score can be given as follows: the study can be rated as “strong” if there are no weak components, “moderate” if there is only one weak‐rated element, or “weak” if there are two or more weak components (Thomas et al. 2004).

## 3 Results

### 3.1 Sample Characteristics

**FIGURE 2:**
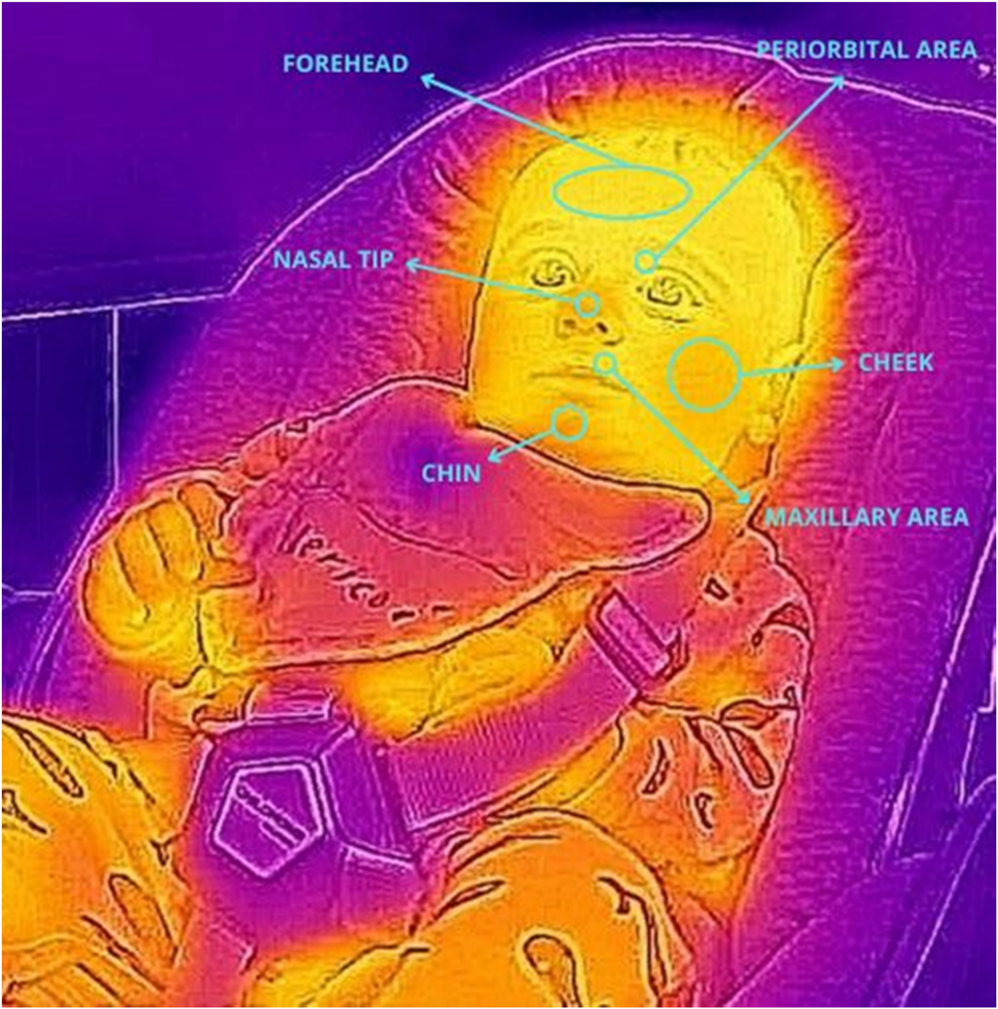
Visual representation of the facial regions of interest (ROIs) employed in the reviewed studies to extract infant temperature.

The selection process (Figure 1) yielded a total of 15 studies with sample sizes ranging from 5 to 186 children. The selected studies were published between 1959 and 2023. Table 1 and Table 2 summarize the characteristics and results of the included studies. Six studies included children between 0 and 2 years old (Tjossem et al. 1959; Mizukami et al. 1990, 1987; Nakanishi and Imai‐Matsumura 2008; Aureli et al. 2015; Ioannou et al. 2021), 8 out of 15 had children between 3 and 6 years old (Tjossem et al. 1959; Hoffner and Cantor 1990; Groer and Howell 1990; Ebisch et al. 2012; Manini et al. 2013; Nicolini et al. 2019; Ganesh et al. 2021; Ohigashi et al. 2023) and 4 studies included children aged between 7 to 12 were included in the study (Hoffner and Cantor 1990; Goulart, Valadão, Delisle‐Rodriguez, Caldeira, et al. 2019; Goulart, Valadão, Delisle‐Rodrigue, Tavares, et al. 2019; Ganesh et al. 2021). All studies had a cross‐sectional design. Only two studies included clinical populations, specifically children with autism spectrum disorder (Ganesh et al. 2021) and children with Moebius syndrome (Nicolini et al. 2019), together with a control group of typically developing children. Most of the reviewed studies assessed temperature changes in infants and children using thermal cameras (*n* = 12), while three studies employed a dermalor (*n* = 1) or a thermistor (*n* = 2). Regions of interest (ROIs, Figure 2) varied quite widely in the reviewed studies, with the nose and the forehead being the most studied ROIs, respectively, in 10 and 9 studies. Studies were quite heterogeneous in terms of experimental procedures employed to assess children's emotion regulation. However, for illustrative purposes, we grouped studies based on assessing thermal affective responses during some kind of social interaction (*N* = 9; Table 1) or using social video–audio stimuli (*N* = 6; Table 2). Social interactions were carried out with a mother, a stranger, or both. The face‐to‐face‐still‐face (FFSF) paradigm, the mishap paradigm, or a separation phase from the mothers were some of the most common procedures . Audio–visual stimuli, such as asking participants to watch video clips to evoke various emotions or stress, were employed with preschool and school‐aged children. Among the reviewed studies, 10 studies focused on evoking negative emotions/distress, one study focused on positive emotions, and four studies on both.

**TABLE 1:** Main characteristics of the reviewed studies employing social interactions to assess emotion regulation.

Study	Sample	Experimental procedure	Emotions	ROIs	Equipment	Sampling rate	Baseline	Key findings
Tjossem et al. (1959)	*N* = 9 2–4 years TDC	Maternal separation	Stress	Dorsum of the non‐dominant hand	Dermalor	0.002–0.003 Hz	NA	↓ temperature during maternal separation
Mizukami et al. (1987)	*N* = 11 6–29 weeks TDC	Mother‐infant play, separation and reunion	Stress	Forehead	TVS4300	NA	Mother‐infant play	↓ temperature during maternal separation
Mizukami et al. (1990)	*N* = 23 8–16 weeks TDC	Maternal separation, interacting with a stranger	Stress	Forehead	TVS1400	NA	Mother–infant play	↓ during maternal separation
Nakanishi and Imai‐Matsumura (2008)	*N* = 6 2–3 months *N* = 10 4–6 months *N* = 6 8–10 months	Mother–infant play	Happiness (laughter)	Forehead, nose, cheeks	TH3104MR	1 HZ	Before laughing	↓ nose temperature during laughter in infants older than 4 months
Ebisch et al. (2012)	*N* = 12 38–42 months TDC	Mishap paradigm	Stress	Nasal tip, maxillary area	FLIR SC3000	50 Hz	Neutral interaction	↓ temperature at the nasal tip and maxillary area after the mishap followed by ↑ during soothing
Manini et al. (2013)	*N* = 14 39–45 months TDC	Mishap paradigm	Stress	Nasal tip	FLIR SC660	15 Hz.	Neutral interaction	↓ temperature at the nasal tip after the mishap followed by ↑ during soothing
Aureli et al. (2015)	*N* = 12 3–4 months TDC	FFSF paradigm	Stress	Forehead, nose	FLIR SC660	50 Hz	Mother‐infant play	↑ nose temperature from baseline to SF and Post‐SF ↑ forehead temperature from the baseline to the final episode
Ioannou et al. (2021)	*N* = 10 2–3 months TDC	Interaction with mother and stranger	Stress	Forehead, chin, cheeks, nose, periorbital region, upper lip	TP8	1 Hz	Neutral interaction	↑ temperature at nose, maxillary area, and forehead with stranger compared to mother
Ohigashi et al. (2023)	*N* = 45 4–6 years TDC	Peeking paradigm	Shame	Forehead, nose	FLIR T650sc	30 Hz	10 s game explanation	↑ nasal temperature after shame

Abbreviations: FFSF, face‐to‐face still face paradigm; NA, not applicable; ROIs, regions of interest; TDC, typically developing children.

**TABLE 2:** Main characteristics of the reviewed studies employing audio–visual stimuli to assess emotion regulation.

Study	Sample	Experimental procedure	Emotions	ROIs	Equipment	Sampling rate	Baseline	Key findings
Groer and Howell (1990)	*N* = 18 4–5 years TDC	Neutral and violent videos	Stress	Right middle finger	Thermistor	0.0208 Hz	1 min before the video	Non‐significant ↓ during violent video
Hoffner and Cantor (1990)	*N* = 186 5–11 years TDC	Adventurous movie sequence	Negative Positive affect	Left pinkie finger	Thermistor	1 Hz	20 s video on nature	↓ temperature during stressful scene in forewarned children ↑ temperature in not forewarned children
Goulart, Valadão, Delisle‐Rodriguez, Caldeira, et al. (2019)	*N* = 28 7–11 years TDC.	5 emotions‐evoking videos	Disgust, fear, happiness, sadness, surprise	Forehead, nasal tip, cheeks, chin, periorbital, perinasal regions	Therm‐App	8.7 Hz	3 s before the videos	↓ temperature at the forehead (disgust, surprise), nasal tip (disgust, fear, happiness), periorbital (happiness, sadness, surprise) and perinasal (disgust, happiness) regions and chin (happiness, sadness, surprise)
Goulart, Valadão, Delisle‐Rodrigue, Tavares, et al. (2019)	*N* = 5 9–11 years TDC	2 emotions‐evoking videos	Happiness, sadness	Forehead, periorbital regions, nasal tip, cheeks	Therm‐App	8.7 Hz	3 s before the videos	Significant variations in all ROIs for happiness vs. baseline and for happiness vs. sadness
Nicolini et al. (2019)	*N* = 9 4–8 years with MBS and N = 15 TDC	1 neutral and 5 emotions‐evoking videos	Sadness, happiness, fear	Nasal tip	FLIR T450sc	5 Hz.	30 s neutral video	↑ temperature during sadness, larger variations from baseline in TDC vs. MBS
Ganesh et al. (2021)	*N* = 50 5–10 years with ASD and N = 50 TDC	3 emotions‐evoking videos	Happiness, anger, sadness	Forehead, eye, nose, cheek	FLIR SC305	NA	NA	↑ temperature in children with ASD vs. TDC in all ROIs

Abbreviations: ASD, autism spectrum disorder; MBS, Moebius syndrome; NA, not applicable; ROIs, regions of interest; TDC, typically developing children.

### 3.2 Using ITI to Measure Emotion Regulation During Social Interaction

The majority of the included studies (*n* = 9) investigated temperature variations during social interactions with either the caregiver (Aureli et al. 2015; Ioannou et al. 2021; Mizukami et al. 1990, 1987; Nakanishi and Imai‐Matsumura 2008; Tjossem et al. 1959) and/or a stranger (Ebisch et al. 2012 Ioannou et al. 2021; Manini et al. 2013; Mizukami et al. 1990; Ohigashi et al. 2023). All studies included samples of typically developing children, with five studies conducted on infants aged 0–23 months (Aureli et al. 2015; Ioannou et al. 2021; Mizukami et al. 1990, 1987; Nakanishi and Imai‐Matsumura 2008) and the remaining four conducted on toddlers aged 2–6 years (Ebisch et al. 2012; Manini et al. 2013; Ohigashi et al. 2023; Tjossem et al. 1959). The sample size of the included studies ranged from a minimum of 6 subjects (Ebisch et al. 2012) to a maximum of 45 (Ohigashi et al. 2023).

The majority (*n* = 8) of the studies focused on assessing thermal affective responses to stress‐inducing social interactions such as maternal separation (Mizukami et al. 1990, 1987; Tjossem et al. 1959), the mishap paradigm, which was introduced by Cole et al. (1992) (Ebisch et al. 2012; Manini et al. 2013), the FFSF paradigm, ideated by Tronick et al. (1978) (Aureli et al. 2015), interaction with a stranger (Ioannou et al. 2021) and, lastly, a peeking paradigm based on Talwar and colleagues work (Talwar et al. 2002) (Ohigashi et al. 2023). Only one study focused on investigating thermal affective response to positive emotionality (i.e., laughter) during social interaction (Nakanishi and Imai‐Matsumura et al. 2008).

Most studies (8 out of 9 reviewed) investigated variations in facial temperature. The most frequently selected ROIs were the forehead (Aureli et al. 2015; Ioannou et al. 2021; Mizukami et al. 1990, 1987; Nakanishi and Imai‐Matsumura 2008; Ohigashi et al. 2023) and the nasal tip (Aureli et al. 2015; Ebisch et al. 2012; Ioannou et al. 2021; Manini et al. 2013; Nakanishi and Imai‐Matsumura 2008; Ohigashi et al. 2023). Additional ROIs were present in three of these studies, such as the maxillary area (Ebisch et al. 2012; Ioannou et al. 2021), the cheeks (Ioannou et al. 2021; Nakanishi and Imai‐Matsumura 2008), and the periorbital area (Ioannou et al. 2021). Only one study (Tjossem et al. 1959) focused on the dorsum of the non‐dominant hand. Notably, this was the only study that measured temperature changes with the use of a dermalor.

Evidence from the pool of articles investigating temperature changes following stress‐inducing social interactions is diverse. Most of them (*n* = 5) revealed temperature decreases during the exposure to the social stressor (i.e., mishap, separation from the caregiver) in the forehead (Mizukami et al. 1990, 1987), nasal tip (Ebisch et al. 2012; Manini et al. 2013), maxillary area (Ebisch et al. 2012), and dorsum of the non‐dominant hand (Tjossem et al. 1959). In addition, this was followed by a temperature increase during the soothing phase in the two studies using the mishap paradigm (Ebisch et al. 2012; Manini et al. 2013). In contrast, studies by Aureli et al. (2015), Ioannou et al. (2021) and Ohigashi and colleagues (Ohigashi et al. 2023) found temperature increases ROIs during (Aureli et al. 2015; Ioannou et al. 2021) and/or after (Aureli et al. 2015; Ohigashi et al. 2023) exposure to the stressor (i.e., maternal still‐face, interaction with a stranger, and shame during the peeking paradigm), particularly at the nasal tip (Aureli et al. 2015; Ioannou et al. 2021; Ohigashi et al. 2023), forehead (Aureli et al. 2015; Ioannou et al. 2021), and maxillary area (Ioannou et al. 2021). Notably, the only study investigating temperature changes during playful interactions (Nakanishi and Imai‐Matsumura 2008) also described temperature decreases in the nasal tip after mother‐induced laughter in children aged 4–10 months but not in 2‐to 3‐month‐old infants.

### 3.3 Using ITI to Measure Emotion Regulation During Exposure to Audio–Visual Stimuli

The remaining six studies explored temperature variations in children during exposure to audio–visual stimuli (Ganesh et al. 2021; Goulart, Valadão, Delisle‐Rodriguez, Caldeira, et al. 2019; Goulart, Valadão, Delisle‐Rodrigue, Tavares, et al. 2019; Gröer and Howell 1990; Hoffner and Cantor 1990; Nicolini et al. 2019). While four of them were conducted exclusively on typically developing children (Goulart, Valadão, Delisle‐Rodriguez, Caldeira, et al. 2019; Goulart, Valadão, Delisle‐Rodrigue, Tavares, et al. 2019; Gröer and Howell 1990; Hoffner and Cantor 1990), the remaining two included samples of children with neurodevelopmental conditions, namely autism spectrum disorder (ASD; Ganesh et al. 2021) and the Möbius syndrome (MBS; Nicolini et al. 2019), providing useful insight about thermal response and emotion regulation in children with altered social functioning (i.e., ASD) and social communication through facial expressions (i.e., MBS).

Studies included preschool (4–5 years; Ganesh et al. 2021; Gröer and Howell 1990; Hoffner and Cantor 1990; Nicolini et al. 2019) and/or school‐aged children (6–11 years; Ganesh et al. 2021; Goulart, Valadão, Delisle‐Rodriguez, Caldeira, et al. 2019; Goulart, Valadão, Delisle‐Rodrigue, Tavares, et al. 2019; Hoffner and Cantor 1990; Nicolini et al. 2019), with sample sizes ranging from 5 (Goulart, Valadão, Delisle‐Rodriguez, Caldeira, et al. 2019) to 186 individuals (Hoffner and Cantor 1990). Audio–visual stimuli consisted in emotion‐inducing videos retrieved from the internet or brief edited movie sequences, that were intended to evoke either distress (Gröer and Howell 1990; Hoffner and Cantor 1990) or primary emotions, the most common being happiness and sadness (Ganesh et al. 2021; Goulart, Valadão, Delisle‐Rodriguez, Caldeira, et al. 2019; Goulart, Valadão, Delisle‐Rodrigue, Tavares, et al. 2019; Nicolini et al. 2019), followed by fear (Goulart, Valadão, Delisle‐Rodriguez, Caldeira, et al. 2019; Nicolini et al. 2019), disgust (Goulart, Valadão, Delisle‐Rodriguez, Caldeira, et al. 2019), surprise (Goulart, Valadão, Delisle‐Rodriguez, Caldeira, et al. 2019), and anger (Ganesh et al. 2021). Analogously to the studies with face‐to‐face social interactions, temperature changes during exposure to emotionally evocative audio–visual stimuli were assessed mainly in the facial area with the use of a thermal camera (Ganesh et al. 2021; Goulart, Valadão, Delisle‐Rodriguez, Caldeira, et al. 2019; Goulart, Valadão, Delisle‐Rodrigue, Tavares, et al. 2019; Nicolini et al. 2019). In these studies, the most chosen ROIs were the nasal tip (Ganesh et al. 2021; Goulart, Valadão, Delisle‐Rodriguez, Caldeira, et al. 2019; Goulart, Valadão, Delisle‐Rodrigue, Tavares, et al. 2019; Nicolini et al. 2019), the forehead and the cheeks (Ganesh et al. 2021; Goulart, Valadão, Delisle‐Rodriguez, Caldeira, et al. 2019; Goulart, Valadão, Delisle‐Rodrigue, Tavares, et al. 2019), followed by the periorbital area (Goulart, Valadão, Delisle‐Rodriguez, Caldeira, et al. 2019; Goulart, Valadão, Delisle‐Rodrigue, Tavares, et al. 2019), the orbital area (Ganesh et al. 2021), the perinasal area, and the chin (Goulart, Valadão, Delisle‐Rodriguez, Caldeira, et al. 2019). Only two studies (Gröer and Howell 1990; Hoffner and Cantor 1990) assessed temperature changes during exposure to audio–visual stimuli by placing a thermistor in either the right middle finger (Gröer and Howell 1990) or the left pinkie finger (Hoffner and Cantor 1990).

Studies that included exclusively typically developing individuals reported diverse findings. Two studies reported significant temperature decreases during exposure to distressing videos (Hoffner and Cantor 1990) and videos eliciting primary emotions such as happiness, sadness, surprise, fear, and disgust (Goulart, Valadão, Delisle‐Rodriguez, Caldeira, et al. 2019). While in the study of Hoffner and Cantor (1990), these changes were detected only in the left pinkie finger of children who were forewarned about the distressing situation, Goulart and colleagues (Goulart, Valadão, Delisle‐Rodriguez, Caldeira, et al. 2019) found these changes in several ROIs according to the emotion evoked by the video: changes in the nasal tip were associated with the presentation of disgust, fear, and happiness‐inducing videos, temperature decreases in the periorbital region and chin were related to the presentation of happiness, sadness, and surprise‐inducing videos, whereas temperature changes in the perinasal region and in the forehead were respectively associated with videos eliciting disgust and happiness and disgust and surprise. Significant variations in facial skin temperature during exposure to videos eliciting happiness and sadness versus baseline were also reported in the study by Goulart, Valadão, Delisle‐Rodrigue, Tavares, et al. (2019), although the direction of changes was not specified. Lastly, the study by Gröer and Howell (1990) found no significant change in skin temperature during exposure to distressing videos depicting violent scenes.

Regarding the two studies including a clinical sample of children with neurodevelopmental conditions, the findings by Ganesh et al. (2021) highlighted a tendency of ASD individuals to display higher skin temperatures at all examined ROIs, regardless of the emotion elicited, compared to typically developing children. Interestingly, the temperature increases in ASD individuals were particularly pronounced when watching anger‐eliciting videos in the forehead, eye, cheek, and nasal tip. Contrarily, the findings by Nicolini et al. (2019) on children with MBS highlighted that, although both clinical and control groups exhibited greater skin temperature in the nasal tip when watching sadness‐inducing videos compared to happiness and fear‐inducing ones, skin temperature changes in MBS individuals were significantly lower than those exhibited by the control group.

### 3.4 Methodological Quality of the Reviewed Studies

Table 3 summarizes the methodological quality of the reviewed studies following the EPHPP Quality Assessment. Overall, 5 out of 15 studies were qualified as moderate (Groer and Howell 1990; Aureli et al. 2015; Nicolini et al. 2019; Goulart, Valadão, Delisle‐Rodriguez, Caldeira, et al. 2019; Ohigashi et al. 2023), the remaining 10 studies were weak according to the 6 components (Tjossem et al. 1959; Mizukami et al. 1990, 1987, Hoffner and Cantor 1990, Nakanishi and Imai‐Mitsumura 2008; Ebisch et al. 2012; Manini et al. 2013; Goulart, Valadão, Delisle‐Rodrigue, Tavares, et al. 2019; Ioannou et al. 2021; Ganesh et al. 2021). In the first element, selection bias, while 8 studies were rated as weak (Mizukami et al. 1990, 1987; Nakanishi and Imai‐Mitsumura 2008; Ebisch et al. 2012; Manini et al. 2013; Goulart, Valadão, Delisle‐Rodrigue, Tavares, et al. 2019; Ioannou et al. 2021; Ganesh et al. 2021), 7 studies were measured as moderate (Tjossem et al. 1959; Groer and Howell 1990; Hoffner and Cantor 1990; Aureli et al. 2015; Nicolini et al. 2019; Goulart, Valadão, Delisle‐Rodriguez, Caldeira, et al. 2019; Ohigashi et al. 2023). According to the study design component, 13 studies were found to be as weak (Mizukami et al. 1990, 1987; Groer and Howell 1990; Hoffner and Cantor 1990; Ebisch et al. 2012; Manini et al. 2013; Aureli et al. 2015; Nicolini et al. 2019; Goulart, Valadão, Delisle‐Rodriguez, Caldeira, et al. 2019; Goulart, Valadão, Delisle‐Rodrigue, Tavares, et al. 2019; Ioannou et al. 2021; Ganesh et al. 2021; Ohigashi et al. 2023) whereas the remaining 2 studies were rated as moderate (Tjossem et al. 1959; Nakanishi and Imai‐Mitsumura 2008). According to the third element, in which confounders were considered, 7 studies were found to be strong (Ebisch et al. 2012; Aureli et al. 2015; Nicolini et al. 2019; Goulart, Valadão, Delisle‐Rodriguez, Caldeira, et al. 2019; Goulart, Valadão, Delisle‐Rodrigue, Tavares, et al. 2019; Ioannou et al. 2021; Ganesh et al. 2021), 3 studies were measured as moderate (Groer and Howell 1990; Manini et al. 2013; Ohigashi et al. 2023) and 5 studies were rated as weak (Tjossem et al. 1959; Mizukami et al. 1987; Mizukami et al. 1990; Hoffner and Cantor 1990; Nakanishi and Imai‐Mitsumura 2008). In the 4th element, the studies were considered if there was a blinding process. All studies were rated as moderate in this element (Tjossem et al. 1959; Mizukami et al. 1990, 1987; Groer and Howell 1990; Hoffner and Cantor 1990; Nakanishi and Imai‐Mitsumura 2008; Ebisch et al. 2012; Manini et al. 2013; Aureli et al. 2015; Nicolini et al. 2019; Goulart, Valadão, Delisle‐Rodriguez, Caldeira, et al. 2019; Goulart, Valadão, Delisle‐Rodrigue, Tavares, et al. 2019; Ioannou et al. 2021; Ganesh et al. 2021; Ohigashi et al. 2023). It should be noted that all studies were coded as strong in the data collection method component (Tjossem et al. 1959; Mizukami et al. 1990, 1987; Groer and Howell 1990; Hoffner and Cantor 1990; Nakanishi and Imai‐Mitsumura 2008; Ebisch et al. 2012; Manini et al. 2013; Aureli et al. 2015; Nicolini et al. 2019; Goulart, Valadão, Delisle‐Rodriguez, Caldeira, et al. 2019; Goulart, Valadão, Delisle‐Rodrigue, Tavares, et al. 2019; Ioannou et al. 2021; Ganesh et al. 2021; Ohigashi et al. 2023). In the last component, the studies were evaluated regarding the withdrawals and drop‐outs, in which 2 studies were measured as weak (Tjossem et al. 1959; Nakanishi and Imai‐Mitsumura 2008), and the remaining 13 studies were found to be moderate (Mizukami et al. 1990, 1987; Groer and Howell 1990; Hoffner and Cantor 1990; Ebisch et al. 2012; Manini et al. 2013; Aureli et al. 2015; Nicolini et al. 2019; Goulart, Valadão, Delisle‐Rodriguez, Caldeira, et al. 2019; Goulart, Valadão, Delisle‐Rodrigue, Tavares, et al. 2019; Ioannou et al. 2021; Ganesh et al. 2021; Ohigashi et al. 2023).

**TABLE 3:** Quality appraisal of the included studies.

Study	A	B	C	D	E	F	Global score
Tjossem et al. (1959)	2	2	3	2	1	3	**3**
Mizukami et al. (1987)	3	3	3	2	1	2	**3**
Mizukami et al. (1990)	3	3	3	2	1	2	**3**
Groer and Howell (1990)	2	3	2	2	1	2	**2**
Hoffner and Cantor (1990)	2	3	3	2	1	2	**3**
Nakanishi and Imai‐Mitsumura (2008)	3	2	3	2	1	3	**3**
Ebisch et al. (2012)	3	3	1	2	1	2	**3**
Manini et al. (2013)	3	3	2	2	1	2	**3**
Aureli et al. (2015)	2	3	1	2	1	2	**2**
Nicolini et al. (2019)	2	3	1	2	1	2	**2**
Goulart, Valadão, Delisle‐Rodriguez, Caldeira, et al. (2019)	2	3	1	2	1	2	**2**
Goulart, Valadão, Delisle‐Rodrigue, Tavares, et al. (2019)	3	3	1	2	1	2	**3**
Ioannou et al. (2021)	3	3	1	2	1	2	**3**
Ganesh et al. (2021)	3	3	1	2	1	2	**3**
Ohigashi et al. (2023)	2	3	2	2	1	2	**2**

*Note:* Labels: A, Selection bias; B, Study design; C, Confounders; D, Blinding; E, Data collection methods; F, withdrawals, and drop‐outs. Quality codes: 1, strong; 2, moderate; 3, weak.

## 4 Discussion

The ability to effectively regulate socio‐emotional states begins to develop early in life and continues to evolve throughout the lifespan. This capacity plays a crucial role in influencing individual's psychological well‐being and mental health outcomes. Investigating developmental trajectories of emotion regulation and identifying the factors that influence them is critical for informing early interventions and promoting healthy developmental pathways.

ITI represents a promising tool for the investigation of early stress and emotion regulation in a non‐invasive and ecological manner, offering insight into dynamic, real‐time processes. However, its application in developmental research remains limited. The current review yielded 15 studies spanning over six decades with sample sizes ranging from 5 to 186 children. The limited number of evidence, together with substantial methodological variations, particularly in infants’ age, study design, experimental procedures, and ROIs analyzed, limits conclusions that can be drawn from the available studies. In what follows, we will summarize the key findings from the reviewed studies, highlighting the potential of ITI in advancing our understanding of emotion regulation early in life. We will also discuss methodological challenges that need to be addressed to enhance the consistency, interpretability, and broader applicability of ITI‐based research in this field.

### 4.1 Wealth of Evidence

The experimental paradigms were broadly categorized into social interactions (*n* = 9) and exposure to audio–visual stimuli (*n* = 6). In both categories, ITI predominantly focused on facial regions, with the nasal tip and forehead emerging as the most frequently studied ROIs. However, the studies varied widely in their choice of additional ROIs and measurement devices, reflecting a lack of standardization. This heterogeneity is typical of emergent scientific methodologies and likely contributes to the variability in findings across studies, highlighting the need for methodological consistency to enhance comparability and reproducibility. Noteworthy, the methodological quality assessment of the reviewed studies revealed that only 5 out of 15 studies were rated as having moderate quality, while the remaining 10 studies were categorized as weak based on the six evaluated components. This highlights a critical need for improved rigor in study design and reporting within this field.

Studies assessing emotion regulation during social interactions (*n* = 9) employed paradigms such as the FFSF, maternal separation, and interaction with strangers or the mishap paradigm. These paradigms primarily elicited negative emotional states such as distress or shame, although one study explored temperature variations occurring during laughter episodes.

Findings suggest that temperature changes in the forehead and nasal tip might reflect SNS‐mediated stress responses, with temperature decreases being observed during the stressful situation (Mizukami et al. 1990, 1987; Ebisch et al. 2012; Manini et al. 2013) and subsequent increases during recovery, reflecting a withdrawal of sympathetic vasoconstrictor tone (Ebisch et al. 2012; Manini et al. 2013). However, contradictory findings were also reported with children's facial temperature increases being observed during the experimental paradigm (Aureli et al. 2015; Ioannou et al. 2021; Ohigashi et al. 2023). In addition to reflecting methodological variations, divergent findings may also point to complex interactions between the sympathetic and parasympathetic branches of the ANS, as well as to the distinct nature of the stressors employed across paradigms. For example, according to the Polyvagal theory, the SNS is typically activated in response to moderate to high levels of threats or challenges (Porges 1995). In contrast, milder stressors or socially engaging experiences—such as those examined in some of the reviewed studies (e.g., Ioannou et al. 2021; Ohigashi et al. 2023)—may predominantly involve PNS withdrawal, without strong sympathetic activation (Porges 2007b). Furthermore, it is also possible that distinct thermal affective responses reflect individual differences in emotion regulation strategies, with SNS‐driven changes being observed more often in individuals who are more prone to defensive mobilization. However, larger, well‐powered studies are needed to reliably identify distinct patterns of thermal responses and associate them with specific emotion regulation profiles.

Interestingly, temperature decreases were also observed during pleasant mother–infant interactions and particularly during laughter episodes in infants older than 4 months (Nakanishi and Imai‐Matsumura 2008). This evidence highlights the complexity of interpreting thermal responses in the context of emotion regulation, and it might be hypothesized that task features should be carefully considered as potential relevant influences on thermal response itself. Likewise, task demands, such as cognitive load or attentional requirements, might also contribute to shaping thermal affective responses, beyond the emotional valence. In this light, a temperature decrease may not exclusively indicate distress but could also reflect general arousal or heightened attentional engagement. In adults, a task‐evoked temperature decrease at the nasal tip has been reported during cognitive tasks (Pinti et al. 2015; Or and Duffy 2007). Conversely, in a small sample of 6 years old children, processing abstract words, as compared to concrete words, was found to be associated with an increase in nasal tip temperature of the nasal tip, which was hypothesized to reflect greater cognitive and social engagement mediated by parasympathetic processes (Paoletti et al. 2022).

Notably, all these studies, except for Ohigashi et al. (2023), were conducted on a small sample size, which significantly limits the robustness and generalizability of the conclusions that can be drawn. Small sample size inherently reduces statistical power, increasing the risk of Type II errors, and limiting the ability to identify subtle or complex relationships. This limitation is particularly important as subtle individual differences in physiological responses can be masked by high variability and low statistical power. Addressing this limitation in future research will require prioritizing larger, adequately powered studies to ensure that findings are statistically robust and generalizable, advancing the field in a meaningful way.

Parallel insights from adult research provide valuable context. Ioannou et al. (2014) showed that proximity and gaze modulate facial thermal responses during interactions. Specifically, facial temperature increased when transitioning from a social to an intimate distance, but not when moving in the opposite direction. In addition, direct gaze was associated with higher facial temperatures than averted gaze. Similarly, Hahn et al. (2012) reported that social contact during interactions increased facial temperature, particularly in the periorbital region, nose, and mouth. Fewer studies have investigated adult facial thermal responses during stressful social interactions. Merla and Romani (2007) found that being observed by unfamiliar people while failing a Stroop task led to a temperature decrease around the mouth and nose tip, with the most pronounced effects observed in participants sensitive to social judgment. Other studies have highlighted temperature increases in the periorbital region (Zhu et al. 2007) and the supraorbital vessels of the forehead (Pavlidis et al. 2002) during mock interrogations for a simulated crime.

Together, these findings from both pediatric and adult populations underline the complexity of interpreting the thermal affective responses to social stimuli. Crucially, such responses may not simply reflect emotional experiences but rather serve as dynamic markers of regulatory processes, particularly in socially demanding contexts. In this vein, facial temperature patterns may indicate attempts to manage emotional arousal as well as co‐regulation efforts. This perspective encourages a more integrative interpretation of ITI data, in which thermal variations are understood as reflecting not only emotional reactivity, but also adaptive regulatory mechanisms shaped also by task demands and social context. Further research is clearly needed to enable deeper exploration of these processes and clarify the context under which specific thermal patterns emerge.

The remaining studies (*n* = 6) explored temperature variations in children during exposure to audio–visual stimuli, with two of them including clinical populations such as children with ASD and MBS. Due to the nature of the experimental paradigm, these studies were performed on older samples of children, from preschool to school age, and examined a wide range of emotions. Among typically developing children, temperature decreases were frequently observed in response to distressing or primary emotion‐inducing videos (Hoffner and Cantor 1990, Goulart, Valadão, Delisle‐Rodriguez, Caldeira, et al. 2019) with wide variability in the ROIs involved. Notably, Goulart and colleagues (Goulart, Valadão, Delisle‐Rodriguez, Caldeira, et al. 2019) reported that different emotional stimuli elicited unique responses across ROIs, though a distinct thermal affective signature could not be identified for each emotion. Overall, happiness, surprise, and disgust were associated with greater thermal variations in the periorbital region, cheeks, and nose when compared to baseline conditions. This pattern suggests that arousal, rather than valence, may play a dominant role in shaping thermal affective responses, aligning with findings in adult populations (Salazar‐López et al. 2015). Evidence from adult studies indicates that valence effects—positive emotions increasing temperature and negative emotions decreasing it—are most pronounced during low‐arousal stimuli. In contrast, high‐arousal stimuli tend to elicit broader thermal responses, irrespective of emotional valence. These findings highlight the need to consider both arousal and valence dimensions when interpreting thermal data, particularly when examining dynamic emotional experiences in children. In clinical populations, findings revealed distinct patterns. Children with ASD demonstrated higher baseline skin temperatures across all examined ROIs compared to their typically developing peers, with more pronounced increases during anger‐eliciting videos (Ganesh et al. 2021). These results suggest heightened physiological arousal in ASD, potentially linked to altered emotional processing mechanisms. Conversely, children with MBS exhibited attenuated temperature changes in response to sadness‐inducing videos compared to control groups (Nicolini et al. 2019). These attenuated responses may reflect differences in the autonomic underpinnings of emotional experience, mainly related to the impairment of facial movement in MBS.

Notably, two studies (Ganesh et al. 2021; Goulart, Valadão, Delisle‐Rodriguez, Caldeira, et al. 2019) employed ITI to classify emotions based on facial skin temperature changes to provide insight for automated tools for emotion recognition. Ganesh et al. (2021) compared autistic and neurotypical children's facial temperatures during emotional stimuli (i.e., happiness, anger, and sadness) and, using a custom convolutional neural network, achieved a classification accuracy of 96%, highlighting the potential of ITI as a supporting diagnostic tool for ASD. Conversely, Goulart, Valadão, Delisle‐Rodriguez, Caldeira, et al. (2019) developed a classification method based on linear discriminant analysis to recognize five emotions (i.e., disgust, fear, happiness, sadness, and surprise) in typically developing children, achieving an average accuracy of 85.25% and a Kappa coefficient of 81.26%. The highest accuracy was observed for disgust (89.88%) and happiness (88.22%), while sadness showed the lowest accuracy (74.70%). These findings underscore the effectiveness of ITI in identifying emotional states and the potential of these approaches for further applications in developmental and clinical research.

Although our review spans a broad age range—from infancy through late childhood—the available evidence does not allow for definitive conclusions about developmental trajectories in thermal responsivity. This limitation is largely driven by the lack of longitudinal or age‐comparative designs. Furthermore, considerable variability in the paradigms used across different age groups has been noted. In infants and toddlers (0–6 years), thermal responses were primarily assessed through social interactions with caregivers or unfamiliar adults. These contexts likely tap into early attachment and interpersonal regulatory mechanisms. In contrast, studies involving older children (7–12 years) predominantly used emotion‐inducing videos, potentially engaging more differentiated and cognitively‐mediated emotional responses. This paradigm heterogeneity may interact with underlying developmental changes in autonomic control and emotion regulation strategies, thereby confounding any straightforward interpretation of age‐related effects in thermal affective responses. From a developmental neuroscience perspective, it is well‐established that the maturation of prefrontal regions and associated regulatory networks influences the expression and modulation of emotional and stress‐related responses (Motzkin et al. 2015; Tottenham 2017). However, how the neurobiological shifts in emotion regulation might reflect in thermal affective responses during emotion‐related tasks remains an important area for future investigation. We recommend that future research adopt developmentally sensitive designs and consistent paradigms across age groups to clarify how maturation shapes thermal responsivity patterns and their underlying mechanisms.

### 4.2 Methodological Issues

Although ITI represents an innovative and potentially useful technique for the investigation of emotion regulation abilities early in life, several methodological issues deserve consideration when designing an ITI study. As skin thermal responses to social challenges could result in small temperature variations of the ROIs examined, it is crucial to exclude that the observed variations are due to either individual, environmental, or technical factors.

#### 4.2.1 Individual Factors

First, participants are requested to avoid the use of any vasomotor substance, such as coffee, tea, alcohol, drugs, tobacco, and sparkling water on the day of the testing, as the intake of these substances would bias the skin thermal pattern (Fernández‐Cuevas et al. 2015). Likewise, moisturizing cream or lotion, sunscreen, and makeup need to be avoided to allow an accurate measure of facial temperature (Robinson et al. 2012). Also, it has been suggested to remove corrective eyewear during the ITI registration, as glass is opaque to infrared light, and to give enough time after removal to allow pressure‐related temperature recovery (Gane et al. 2011). Second, exercise can lead to increased energy expenditure and affect skin temperatures up to 14 h post‐exercise, thus exercise is an additional individual factor to account for (Fernández‐Cuevas et al. 2015). Third, circadian rhythm can influence body temperature. Thus, the experiment should be conducted around the same time of day and season (Fernández‐Cuevas et al. 2015). Beyond these physiological considerations, psychological and emotional states represent individual factors that can significantly influence individuals’ thermal responsivity. Researchers should consider assessing participants' emotional state or mood immediately prior to testing, as this might influence subsequent physiological reactions. Furthermore, individual differences in temperament, attachment style, and early exposure to adverse environmental stressors can shape how individuals perceive and respond to stressful and emotional stimuli (Groh and Narayan 2019; Nazzari et al., 2022; Talge et al. 2008; Nazzari et al., 2023), potentially leading to distinct thermal signatures. Accounting for these factors can help explain variability in ITI findings and refine our understanding of emotion regulation dynamics. Last, there are some aspects related to typical cutaneous thermoregulations that might constitute exclusion criteria for participation in an ITI study, such as peripheral neuropathy, micro and macroangiopathies, connective tissue diseases, and psychophysiological disorders (Ioannou, Gallese, et al. 2014). Notably, some medical conditions possibly influencing autonomic reactivity as well as skin temperatures might be undiagnosed in young participants, possibly introducing unmeasured variability in data. This underscores the need for thorough participant screening and careful interpretation of findings in pediatric samples.

#### 4.2.2 Environmental Factors

The temperature and humidity of the experimental room need to be strictly controlled, with a temperature ranging between 18° and 23° being recommended by the International Academy of Thermology (IACT) guidelines (IACT 2002) and a humidity range between 40% and 70% (Fernández‐Cuevas et al. 2015). No direct ventilation on participants, as well as direct sunlight, is permitted. A thermal acclimatization phase during the 15 min before the experiment is highly recommended, as the skin temperature is continuously adapted in response to the environmental conditions. It is also important to note that while temperature changes in response to an emotional/stressful stimulus can be rapidly detected (15–20 s), it is still unclear how much time is needed for the facial temperature to recover to baseline values after a rise. Animal models suggest that this time depends on the ROIs analyzed (Vianna and Carrive 2005). For example, rodents’ backs, heads, and bodies take around 60–75 min to return to baseline values, whereas the eyes, tails, and paws take between 10 and 15 min (Travain and Valsecchi 2021). These findings raise concerns regarding the interpretation of thermal response to short‐duration experimental paradigms that are often used in developmental research. Without adequate acclimatization before the procedure or sufficient recovery time post‐stimulus, the resulting thermal data may reflect transient physiological responses, complicating the identification of stimulus‐specific effects. Further studies in humans across development are needed to better characterize these dynamics and inform optimal experimental design.

#### 4.2.3 Technical Factors

To date, no protocol has been created for ITI studies measuring facial thermal variations during emotion regulation tasks. However, a number of technical factors are known to influence ITI measurement and need to be accounted for. First, the distance between the camera and the participant depends on the size of the ROIs and on the camera's optics. Furthermore, the camera must be placed as much as possible orthogonal to the plane of the region of investigation, to maximize the reading of the flux of thermal energy. It is also important not to obstruct the view of the camera. In studies involving young participants, such as infants, unconstrained movements are clearly an issue. They might lead to motion artifacts, where the selected ROIs may shift or become distorted, causing inaccurate temperature readings. Furthermore, partial occlusions, for instance, when an infant brings their hand to their face or turns away, can lead to missing or unreliable thermal data. A manual tracking of ROIs during post‐processing is often employed to mitigate noise related to infants’ rapid head movements. Frames, where faces are obstructed or marked, head‐turning prevents accurate ROI selection, should be discarded, and the percentage of discarded frames should be reported in the manuscript. However, manual tracking of the signal is resource‐consuming and prone to human error, underscoring the importance of developing and implementing robust automated tracking algorithms. Such algorithms could significantly improve the accuracy and reliability of ITI data in dynamic pediatric contexts by continuously identifying and tracking ROIs, even with minor movements or brief occlusions. Furthermore, maternal touching of the face of the child during the ITI recordings might confound the assessment and should thus be avoided. In addition, while in the reviewed studies, temperature measurements were extracted continuously, frame acquisition rates varied substantially among studies. Generally, higher sampling rates allow for more efficient tracking of movement, provide more details about the temporal latency of thermal variations, and allow for the possibility of discarding frames disturbed by participants’ movements. Last, in order to investigate autonomic activation in response to social or emotional challenges, it is important to select an appropriate baseline condition. Different types of baselines have been employed in ITI studies according to the study aims, as reviewed here. Considering that there is no gold standard regarding the selection of a suitable baseline, it is crucial for researchers to reach an inter‐study consensus on the most adequate autonomic “starting point” for reliable experimental comparisons and generalizability of the results across studies. Given the relatively novel application of ITI in developmental psychology, it is also important to encourage the development and open publication of detailed protocol papers (e.g., Hinrichs et al., 2025) and to make processing pipelines and algorithms openly accessible (e.g., Nazzari et al., 2025) Such transparency will foster greater standardization, reproducibility, and comparability across studies, which is essential for the rigorous progression of the field.

### 4.3 Future Directions and Conclusions

As interest in understanding mechanisms underlying early emotion regulation grows, there is a pressing need for methods that are both sensitive and ecologically valid, enabling the assessment of infants’ physiology in real‐world interactive settings. While traditional physiological measures, such as heart rate and skin conductance, are increasingly accessible and widely used in pediatric research (Nazzari, Reali, et al. 2022; Quadrelli et al. 2019; Nava et al. 2016; Natale et al. 2014; Quadrelli et al. 2021), they come with some limitations. For instance, these metrics often rely on contact‐based sensors, which require participants’ compliance and a considerable amount of time for accurate application. Such constraints can be particularly challenging when studying infants or clinical populations, where compliance and tolerance to sensors may be limited. ITI is an emerging contact‐free technique increasingly employed to investigate the biological underpinnings of emotion and stress regulation in adults. Thanks to its non‐invasiveness and contact‐free nature, ITI provides a unique opportunity to study natural social interactions without interfering with spontaneous behaviors in an ecologically valid setting. In this review, we systematically examine existing applications of ITI to the study of emotion regulation early in life. Albeit preliminary, existing studies extend our knowledge in at least two significant ways.

First, available studies indicate that as early as 2 months of age, facial thermal variations are sensitive to social stimuli and can capture subtle autonomic activation associated with emotional states. Although more studies are needed to identify a clear pattern of thermal affective responses, results suggest that ITI might be a sensitive measure of physiological arousal and emotion regulation processes, even in populations where traditional methods pose practical challenges, such as infants and young children. However, it is important to acknowledge that existing literature is limited by poor methodological quality and small sample sizes. Therefore, larger and more rigorous studies are needed to provide stronger empirical support for the use of ITI early in life. Notably, studies with adults have emphasized the influence of both individual and contextual factors on thermal affective responses to social stimuli (e.g., Ioannou et al. 2014; Hahn et al. 2012). These variables should be examined in future studies and hopefully clarify the emerging inconsistencies in the literature.

Second, ITI has shown promise in studying atypical developmental trajectories. Although only two studies examined clinical populations, they both revealed distinct thermal response patterns in children with ASD (Ganesh et al. 2021) and MBS (Nicolini et al. 2019) compared to control children, shedding light on how emotion regulation mechanisms may differ in these groups. Such successful applications highlight the utility of ITI as a tool for exploring variability in physiological responses in especially vulnerable samples. For example, ITI might be a useful option in samples of children with high skin sensitivity, such as children with ASD or children with somatic diseases, such as atopic dermatitis, or conditions related to skin fragility, such as very small, premature babies. In these cases, contact‐free techniques like ITI, which do not require the application of electrodes or other devices on the skin, may offer a safer and more comfortable alternative. Furthermore, the development of small, portable, and affordable ITI devices holds the potential to bridge the gap between the controlled environment of a laboratory and real‐life natural settings.

Nevertheless, it is important to emphasize that findings from current studies are somewhat conflicting, and methodological quality remains an issue. Future research should prioritize larger, adequately powered samples and standardized protocols to better capture the complex interplay of individual, contextual, and methodological factors influencing thermal responses to emotional stimuli.

Key considerations include accounting for the range of individual and environmental factors that may interfere with thermal assessments, as well as technical issues such as distance, motion, and angle variations. Likewise, further empirical research should establish what constitutes an appropriate baseline for thermal measurements. Moreover, the advancement of software for the automatic extraction of thermal data from ROIs will enhance the reliability and widespread adoption of ITI. Although all the included studies were cross‐sectional, longitudinal research is needed to investigate how thermal responses to emotional stimuli develop over time and their potential implications for long‐term developmental outcomes. Work by Nakanishi and Imai‐Matsumura (2008) compared small groups of infants at different ages and provided preliminary evidence suggesting that age‐related differences in thermal affective responses may exist. Further longitudinal studies would help clarify the trajectory of these responses and their significance in early emotional and physiological development. Last, more studies are needed that correlate thermal measurements with other well‐established physiological and behavioral indicators of stress regulation in infancy. Addressing methodological inconsistencies, expanding the scope to diverse populations, and incorporating longitudinal designs will be essential to fully realize the potential of ITI. By leveraging its strengths, ITI research can advance our understanding of how early physiological responses shape emotional and social development, offering valuable insights into the foundations of emotion regulation and its disruptions, ultimately guiding effective interventions.

## Conflicts of Interest

The authors declare no conflicts of interest.

## Data Availability

This review article does not report any original datasets. All data analyzed and discussed were obtained from previously published studies, which are appropriately cited within the text. Readers can access these data through the original publications and sources referenced in the manuscript. No new data were generated or analyzed during the preparation of this work.
